# Reducing Blood Culture Contamination Rates: Introduction of a Combined Education and Skin Antisepsis Intervention

**DOI:** 10.1099/acmi.0.000806.v3

**Published:** 2024-07-02

**Authors:** Niamh Mullane, Niall O'Mara, Darragh Coffey, Aine Connolly, Isabelle O'Callaghan, Deborah Kelly, Deirdre Broderick, Caitriona Hickey

**Affiliations:** 1 Department of Clinical Microbiology, Cork University Hospital, Wilton, Cork, Ireland

**Keywords:** blood culture contamination, education, intervention, simulation training, skin antisepsis

## Abstract

**Background.** Blood culture contamination (BCC) is an important quality concern in clinical microbiology as it can lead to unnecessary antimicrobial therapy in patients and increased workload for laboratory scientists. The Clinical Laboratory and Standards Institute recommend BCC rates to be <3 % and recently updated guidelines have set a new goal of 1 %. The aim of this project was to design and implement interventions to reduce BCC rates at our institution.

**Methods.** We introduced a combined education and skin antisepsis intervention in a large Model 4 academic teaching hospital in the South of Ireland. BD ChloraPrep skin antisepsis applicators (2 % chlorhexidine gluconate/70 % isopropyl alcohol), licensed for use for blood culture specimen collection, were introduced, replacing Clinell (2 % chlorhexidine gluconate/70 % isopropyl alcohol) wipes. In addition, a multimodal education programme was designed and delivered. This consisted of a video demonstrating the recommended blood culture specimen collection technique using the new applicators as well as simulation training for all interns. The video was uploaded to the intranet as an educational resource available to all staff.

**Results.** The interventions were implemented in July 2022 and BCC rates pre- and post-intervention were calculated. The average BCC rate for the 12 months preceding the intervention (July 2021 to July 2022) was 2.56 % with highest rates in the Emergency Department. This compared to an average rate of 2.2 % in the 12 months post-intervention (July 2022 to July 2023). In comparing the two rates the reduction in BCC rates between the two periods was not statistically significant (*P*=0.30).

**Conclusion.** Overall BCC rates reduced but the difference between the two periods did not reach statistical significance. The resource-intensive nature of providing regular and timely feedback of contamination rates and the larger impact of in-person education and training over virtual modalities may explain the modest reduction. Further investments in these areas, particularly in the Emergency Department, will be necessary to further reduce rates in line with new recommendations.

## Data Summary

The authors confirm all supporting data and protocols have been provided within the article.

## Introduction

Blood culture contamination (BCC) is an important quality concern in clinical microbiology. Contamination occurs when a micro-organism that is not actually present in the patients’ bloodstream is introduced into the culture during sample collection and therefore is not causing infection [1]. Contaminated blood cultures have significant ramifications for patients as well as the microbiology laboratory which have been well documented in the literature [1,3].

BCC can lead to inappropriate antibiotic treatment [4] which has detrimental effects on individual patient care as well as contributing to the global burden of antimicrobial resistance through inappropriate antibiotic use [5]. Processing blood cultures is a time-consuming task for microbiology scientists and contaminated blood cultures add significant workload and result in poor utilization of laboratory services [6]. This is of relevance in the current climate with critical shortages of laboratory scientists and an ever-increasing demand for diagnostic testing and rapid turnaround times [7]. Healthcare systems are affected too, as BCC results in longer hospital stays and unnecessary treatment all which have financial implications [1].

The leading causes of BCC are improper techniques when collecting the blood sample and inadequate disinfection of the skin. Errors in these crucial steps can result in bacteria present on the skin surface being transferred into the blood culture leading to a false positive result [8,9]. Several studies have shown the benefit of education on blood culture specimen collection technique through a combination of educational videos as well as simulation training [6,10,12]. Currently in our hospital there is no mandatory education or training on blood culture specimen collection technique and the product used for skin antisepsis is not licensed for use for blood cultures.

The Clinical Laboratory and Standards Institute (CLSI) produces guidelines concerning blood culture best practice. There has been a long-standing recommendation to maintain BCC rates of less than 3 % and this is the benchmark used by many institutions [13,14]. Our intervention coincided with the recent update to CLSI guidance for blood culture best practice. The April 2022 guideline has re-emphasized that laboratories should be able to achieve contamination rates ‘substantially lower than 3 %’ and, furthermore, ‘when best practices are followed, a target contamination rate of 1 % is achievable’ [15]. Literature has shown that BCC rates frequently exceed the benchmark of 3 % with several studies showing the highest rates of BCC in the Emergency Department (ED) [13,16, 17]. The objective of this intervention was to design and implement a combined education and skin antisepsis intervention with a goal of reducing BCC rates.

## Methods

The intervention took place in a large Model 4 academic teaching hospital. Interventions were implemented in July 2022 and contamination rates were reviewed for 12 months pre- and post-interventions. All positive blood culture data were reviewed by the clinical microbiology team and contaminated blood cultures were identified. Contamination rates were expressed as a percentage of the total number of blood cultures processed. This is an established process within the laboratory quality management framework, performed on a quarterly basis. To calculate the BCC rate, several criteria were used to determine whether the organism in blood culture represented the cause of a clinically significant infection or a false positive result due to contamination. These included the identity of the organism, the number of positive blood culture sets, the number of positive bottles within a set of blood cultures and quantity of growth, in addition to clinical information obtained through liaising with the clinical team [18].

A systematized literature review was conducted to identify evidence-based interventions and implementation strategies which have been shown to successfully reduce BCC rates. As BCC is generally the result of multiple factors, the evidence supports bundle interventions which use several strategies to achieve a reduction in contamination rates [11,13].

### Skin antisepsis intervention

Products containing 2 % chlorhexidine with 70 % isopropyl alcohol are recommended for skin antisepsis prior to the collection of blood for culture [19]. Commercially available products were reviewed by the Infection Prevention Control committee and subsequently the Drug and Therapeutics committee, and ChloraPrep (BD) skin antisepsis applicators (2 % chlorhexidine gluconate/70 % isopropyl alcohol) [20], licensed for blood culture collection, were introduced to replace the previously used Clinell wipes (2 % chlorhexidine gluconate/70 % isopropyl alcohol). The local policy and procedure document on blood culture specimen collection was updated to reflect the change in skin antisepsis product.

### Education intervention

A video demonstrating blood culture specimen collection technique using the new skin antisepsis applicators was shown to all new interns and uploaded to the intranet as an educational resource available to all staff. Five simulation training sessions were then delivered over a 1 week period to 120 interns. These sessions allowed for practical application of the blood culture specimen collection procedure with supervision and feedback. Timing the project implementation with the annual non-consultant hospital doctor changeover in July was an opportunistic way of incorporating education sessions into the hospital induction training programme.

### Statistical analysis

Data were analysed using SPSS software (Version 27, IBM). BCC rates were calculated per month. Mean BCC rates for the 12 months pre- and post-intervention were compared using Student’s t-test. A *P*-value of <0.05 was considered statistically significant.

## Results

**Fig. 1.**
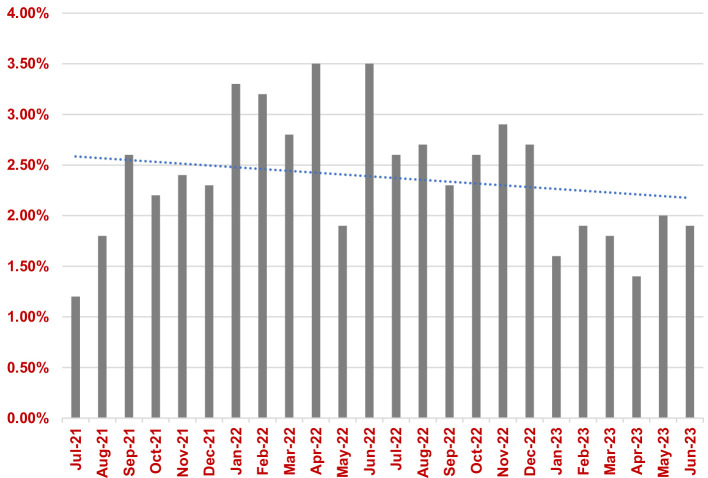
Trend in monthly BCC rates for the 12 months pre- and post-intervention.

All positive blood cultures from July 2021 to July 2023 were reviewed by the clinical microbiology team. In the 12 months pre-intervention (July 2021 to July 2022), 16  404 blood cultures were processed of which 414 were contaminated resulting in an average BCC rate of 2.56 %, 95 % confidence interval (CI) [2.09, 3.02]. In the 12 months post-intervention (July 2022 to July 2023), 19 915 blood cultures were processed of which 430 were contaminated resulting in an average BCC rate of 2.2 %, 95 % CI [1.89, 2.51]. The reduction in BCC rates between the two periods was not statistically significant (*P*=0.30). Fig. 1 shows the trend in monthly BCC rates for the 12 months pre- and post-intervention.

BCC rates were also reviewed by clinical area. The highest rates of BCC were in the ED with a BCC rate of 4.02 % pre-intervention and a BCC rate of 3.8 % post-intervention (Table 1). ED BCC rates were also analysed after excluding samples from the paediatric ED as paediatric samples are inherently technically more challenging to obtain and prone to contamination [21]. The pre-intervention BCC rate in the adult ED was 3.8 % and post-intervention BCC rate was 3.4 %.

**Table 1.** Comparison of ED BCC rates pre- and post-intervention

	Total No. of Blood Cultures Processed	No. of Contaminated Blood Cultures	BCC rate (%)
Pre-intervention(July 2021 to July 2022)	6 690	269	4.02
Post-intervention(July 2022 to July 2023)	7 428	280.4	3.8

## Discussion

Blood cultures are one of the most critical investigations performed in the microbiology laboratory and yet contamination rates are widely reported as exceeding the CLSI recommendations [22,23]. This is despite a significant body of evidence detailing the negative implications of BCC ranging from the impact on patient safety through unnecessary antimicrobial therapy, to the workload placed on the microbiology laboratory as well as the hospital through prolonged hospital admissions [1,6]. While this study did achieve a reduction in BCC rates post-implementation of ChloraPrep (BD) skin antisepsis applicators and introduction of a multi-modal education programme, the reduction in BCC rates did not reach statistical significance.

Our intervention has highlighted the challenges with trying to reduce BCC rates. Prior to this study there was no mandatory education or training on the blood culture specimen collection procedure in our institution. Despite the successful implementation of an education programme, there was only a modest decrease in BCC rates. Education programmes and particularly simulation training are resource-intensive and in our experience due to the constant staff turnover in teaching hospitals, education is an ongoing requirement. Delivering sustainable change in challenging circumstances with limited resources requires an informed understanding of people and culture [24]. Commitment to change has been proposed to arise for several reasons with affective commitment to change, that is staff wanting to change because they understand the associated benefits, being associated with the highest level of support [25,26]. Providing education on the impact of BCC as well as the cost savings associated with reducing BCC would be powerful tools to promote engagement. The education programme implemented in this study has provided a good starting point which can be expanded upon.

We found that the ED had the highest rates of BCC and this is in concordance with findings from other studies investigating BCC rates [13,16, 17]. A multitude of reasons have been identified that explain the association between BCC and the ED environment. The ED is the most common route of admission to the hospital and where initial investigations such as blood cultures are performed, so it accounts for a large volume of the blood cultures processed. It is a fast-paced environment with a high turnover of patients as well as staff and a prospective study highlighted that environmental contamination contributed to BCC rates [27]. There is also a tendency to draw samples from intravenous cannulas to improve efficiency despite this not complying with best practice [28]. The ED also provides care for critically ill patients requiring resuscitation and in these stressful circumstances collecting cultures using an aseptic technique can be less of a priority [13].

Overcrowding is perhaps the most challenging factor which is specific to the ED setting [13] and an ongoing issue in our institution. One study used a scoring system for overcrowding and was able to show that ED overcrowding was independently associated with BCC [29], and another retrospective study of ED blood cultures found that the environment contributed to poor culture collection technique [17]. Poor infrastructure and overcrowding create challenges for staff working in the ED. While we demonstrated a decrease in ED BCC rates post-intervention, the rates remained >3 % even on analysis excluding paediatric samples, which is higher than the CLSI recommendations and the overall average BCC rates for our institution. This underpins the need for dedicated education and training interventions in this area.

A strength of our intervention was the use of multi-modal educational interventions to provide training on blood culture specimen collection technique as the literature supports the use of a combination of educational tools [10,30, 31]. These included a video which was made available to all staff as well as simulation training for all new interns. Including hands-on simulation training has been shown to be beneficial rather than solely relying on virtual modalities [31,32]. We incorporated the simulation training sessions into the hospital induction training programme which is mandatory for all new interns. Therefore, we were able to achieve 100 % attendance at these sessions. Timing the intervention with the non-consultant hospital doctor changeover was effective as the perpetual change of junior staff has been identified as a barrier to reducing BCC rates [33]. ChloraPrep (BD) skin antisepsis applicators were successfully implemented into practice in all clinical areas. Another strength was that all positive blood cultures were rigorously reviewed by one of the clinical microbiology doctors to ensure reporting of contamination and subsequent BCC rate calculations were accurate.

There were several limitations to our intervention. While the video demonstrating the blood culture specimen collection procedure was made available to all staff, due to the resource-intensive nature of simulation training, this was only provided to new interns rather than being offered to all staff responsible for blood culture collection. While providing simulation training to all staff would not have been feasible, the study results have highlighted the benefit of providing in-person training. We suggest focusing available resources on areas with the highest BCC rates such as the ED. Another consideration would be to provide simulation training sessions for interns on a quarterly or biannual basis as interns generally rotate jobs on a 3 monthly basis and often rotate between hospitals.

The literature has shown that providing feedback on BCC rates can be an excellent way to engage staff [22]. While BCC rates are monitored on an on-going quarterly basis in our institution, there is no pathway established to provide feedback directly to clinical areas. While the financial ramifications associated with BCC due to unnecessary treatment and longer hospital stays have been well documented in the literature [1], a financial analysis was not performed in this study, so we were unable to highlight the cost of BCC and the financial impact of reducing BCC rates.

## Conclusion

As multiple factors contribute to BCC, reducing contamination rates requires significant resources and commitment [11]. Despite the challenges, tackling this problem is more important than ever due to the concerning shortages of laboratory scientists and increasing pressure on microbiology laboratories [7]. Furthermore, BCC rates are a key quality indicator and constant effort is required to improve patient safety and management.

Introduction of ChloraPrep (BD) skin antisepsis applicators and a multimodal education programme had a positive impact and timing blood culture training with the annual non-consultant hospital doctor changeover was a useful tactic to engage new staff. Reassuringly BCC rates in our institution were less than 3 % which has been the long-standing benchmark used by many institutions [13,14], but further efforts will be required to achieve a contamination rate of 1 % which updated CLSI guidelines now advocate aiming for [15].

Achieving change in the healthcare system is complex and there has been increasing awareness that the process of change is as important as its content [34]. The resources required to provide simulation training should be optimized and we propose this training should be provided for new staff but also for high-risk areas on an ongoing basis guided by local BCC rates. While BCC rates are under regular surveillance by microbiology departments, there should be increased focus on creating pathways to provide feedback directly to clinical areas on their BCC rate to incentivize staff and promote engagement.

## Abbreviations

BCC: blood culture contamination
CI: confidence interval
CLSI: Clinical Laboratory and Standards Institute
ED: emergency department
